# Internally Filled Fellowship Positions in Obstetrics and Gynecology Before and After 2020

**DOI:** 10.1097/og9.0000000000000107

**Published:** 2025-08-14

**Authors:** Hannah D. McLaughlin, Monica J. Janke, Shitanshu Uppal, Celeste Roberts, Bryan Aaron, Ryan J. Spencer, Jean H. Siedel, Christopher X. Hong

**Affiliations:** Division of Gynecologic Oncology and the Division of Gynecology, Department of Obstetrics and Gynecology, Michigan Medicine, Ann Arbor, and Undergraduate Education, Michigan State University, East Lansing, Michigan; and the Division of Gynecologic Oncology, Department of Obstetrics and Gynecology, University of Wisconsin, Madison, Wisconsin.

## Abstract

**OBJECTIVE:**

To evaluate the proportion of internally filled obstetrics and gynecology fellowship positions before and after 2020.

**METHODS:**

This is a retrospective cohort study of filled training positions within obstetrics and gynecology fellowships to measure the likelihood of internally filled positions compared with externally filled positions 3 years before (2017–2019) and after (2020–2022) the transition to virtual interviews. All positions within gynecologic oncology, maternal–fetal medicine, reproductive endocrinology and infertility, and urogynecology and reconstructive pelvic surgery were included and divided into two match year cohorts. The primary outcome was proportion of internally filled positions. Univariable and multivariable logistic regression assessed the marginal effects of time on the type of filled position.

**RESULTS:**

A total of 1,910 filled positions were identified (gynecologic oncology n=453, maternal–fetal medicine n=805, reproductive endocrinology and infertility n=340, urogynecology and reconstructive pelvic surgery n=312), with 21.4% filled internally (409/1,910). Proportions were similar before and after 2020 (21.3% [190/892] vs 21.5% [219/1,018], *P*=.91). Maternal–fetal medicine had the highest proportion of internally filled positions (26.1% [210/805]), followed by reproductive endocrinology and infertility (22.6% [77/340]), urogynecology and reconstructive pelvic surgery (17.0% [53/312]), and gynecologic oncology (15.2% [69/453]). There was no significant change in the proportion of internally filled positions over time (adjusted marginal change [aMC] 0.2%/year, 95% CI, −0.9 to 1.3%) or between cohorts (delta aMC 2.8%, *P*=.15).

**CONCLUSION:**

Despite the widespread use of virtual interviews, the proportion of internally filled fellowship positions was similar before and after 2020 for all subspecialties, combined, and when assessed individually. This information provides reassurance to program directors and upcoming applicants that the interview format change has not affected internally filled positions.

## Abstract 2

The proportion of internally filled fellowship positions was similar before and after 2020, despite the abrupt transition to virtual interviews.

Before the coronavirus disease 2019 (COVID-19) pandemic, most obstetrics and gynecology fellowship interviews were performed in person, like many other residency and fellowship specialties. At the onset of the pandemic, however, interviews abruptly transitioned from in-person to virtual.^1–4^

Although in-person interviews allow face-to-face interaction and socialization, the benefits of virtual interviews include reduced costs for applicants, less time away from clinical duties, more efficient interview days, and more equity in the interview process.^3,5–10^ Without in-person interviews and an opportunity for direct interaction, it is conceivable that programs may be more likely to accept a candidate outside of the match, when a fellowship position is filled without participation in the National Resident Matching Program (NRMP)—often with a trainee from the fellowship's own institution. Moreover, programs and candidates may rank each other more highly on their rank order lists if they have prior direct experience with one another, which may disadvantage candidates from residency programs without an affiliated fellowship. Although temporal changes in internally filled residency and fellowship positions have been studied in other subspecialties, research is lacking for obstetrics and gynecology subspecialties.^11–15^

The objective of our study was to evaluate the proportion of internally filled obstetrics and gynecology fellowship positions before and after 2020, which coincides with the abrupt adoption of virtual interviews. We hypothesized that there would be a higher proportion of internally filled fellowship positions after 2020, coinciding with increased use of virtual interviews. With this information, we aim to better understand the influence of virtual interviews and provide insight to applicants and programs involved in the fellowship interview process.

## METHODS

We performed a retrospective cohort study of filled training positions within obstetrics and gynecology subspecialty fellowships to measure the likelihood of internally filled positions compared with externally filled positions in the 3 match years before (2017–2019) and after (2020–2022) the transition to virtual interviews during the COVID-19 pandemic. All filled training positions in gynecologic oncology, maternal–fetal medicine, reproductive endocrinology and infertility, and urogynecology and reconstructive pelvic surgery (formerly female pelvic medicine and reconstructive surgery) within active fellowship programs accredited by the Accreditation Council for Graduate Medical Education (ACGME) were identified and included in this analysis. This encompasses fellowship positions filled within and outside of the NRMP, fellowship programs that were newly accredited or underwent a change in accreditation status during the study period, and military-based training programs.

There was no data repository of fellows who filled fellowship training positions for the subspecialties included in this study. To compile such a repository, all subspecialty fellowship programs and positions accredited by the ACGME within the study period were first identified by using the ACGME’s List of Programs by Specialty search tool to ensure a comprehensive collection of all trainees who filled fellowship training positions. Trainees at each fellowship training program with a fellowship fill year (ie, match year) from 2017 through 2022 were then identified through fellowship program websites, communication with fellowship program coordinators and directors, professional networking platforms, and social media platforms. In cases where information regarding a fellowship position for a specific year was unattainable, data were gathered through communication with identified fellows from adjacent years. Trainees were included if they filled a fellowship position regardless of whether their residency program had an affiliated fellowship program. To confirm that each identified trainee was a practicing obstetrician-gynecologist, their profiles were cross-referenced with the National Plan and Provider Enumeration System database to validate a corresponding National Provider Identifier number.

To assess the completeness of the data set, we conducted a comparative analysis between the number of filled fellowship positions with identified trainees in our data set and the total number of filled positions as reported by the ACGME for the academic years 2020–2021, 2021–2022, and 2023. These academic years correspond to match years 2017–2019, 2018–2020, and 2021–2023 for 3-year training positions, which comprise nearly all fellowship positions in this study. The analysis was conducted using academic years, because a comparison of individual match years is limited by the nature of the data provided through the ACGME's data-accreditation system.

Positions within fellowship programs were categorized by whether they were filled by an internal or external trainee. This classification was performed by identifying the residency and fellowship training programs for each trainee in a filled position that used the unique program identification number assigned by the ACGME. A position was considered internally filled if it was secured by a trainee from a residency program that also serves as the core program for the fellowship or has a common sponsoring hospital, as per ACGME records. Internally filled positions encompass those filled both inside and outside the NRMP. Filled training positions were divided into two match year cohorts: 1) before 2020, which included the match years 2017 through 2019; and 2) after 2020, which included the match years 2020 through 2022.

The overall proportion of internally filled positions was calculated. To examine the effects of time on the likelihood of whether positions are internally or externally filled, multivariable logistic regression analysis was used to assess the marginal effects of time, with years considered as a continuous variable. This approach allowed for the evaluation of the trend, or marginal change (MC), in the proportion of internally filled positions. The models were adjusted for subspecialty and accounted for clustering at the fellowship program level to address the nonindependence of observations within programs (ie, certain fellowship programs are more likely than others to fill positions with an internal candidate). A χ^2^ test was performed to evaluate differences in the proportion of internally filled positions between the two cohorts.

To assess whether the year 2020, which marked the transition from in-person to virtual interviews, affected the trends in filling positions internally over time, interaction terms were included for the two cohorts in the analysis. This approach enabled us to evaluate the potential influence of the significant change in interview format on the likelihood of a whether a fellowship position would be filled by an internal trainee. Considering the potential differences in the proportion of internally filled positions across the four subspecialties, subanalyses for each subspecialty were planned and conducted.

Statistical analysis was performed by using RStudio and Stata. *P*<.05 was considered statistically significant. The University of Michigan IRB deemed this study not subject to regulation because it does not involve human subjects research (HUM00237885).

## RESULTS

A total of 1,910 filled fellowship positions from 2017 to 2022 were identified across the four obstetrics and gynecology subspecialties (gynecologic oncology n=453, maternal–fetal medicine n=805, reproductive endocrinology and infertility n=340, urogynecology and reconstructive pelvic surgery n=312). In comparing the number of filled fellowship positions with trainees identified in this study with the ACGME-reported number of filled positions, our data set captured nearly all reported filled fellowship positions for academic years 2020–2021 (96.5% [892/924]), 2021–2022 (98.3% [939/955]), and 2022–2023 (99.7% [981/984]).

**Fig. 1.**
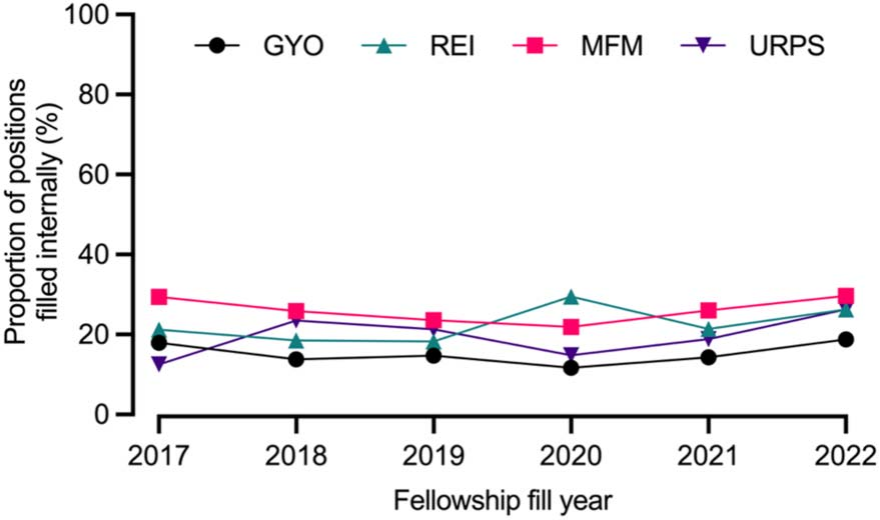
Trends in internally filled positions by subspecialty. GYO, gynecologic oncology; REI, reproductive endocrinology and infertility; MFM, maternal–fetal medicine; URPS, urogynecology and reconstructive pelvic surgery.

Over the study period, the overall proportion of internally filled positions was 21.4% (409/1,910), with similar proportions observed before and after 2020 (21.3% [190/892] vs 21.5% [219/1,018], *P*=.91) (Table 1). Nonetheless, substantial variation was evident among the subspecialties, with maternal–fetal medicine recording the highest proportion of internally filled positions (26.1% [210/805]), followed by reproductive endocrinology and infertility (22.6% [77/340]), urogynecology and reconstructive pelvic surgery (17.0% [53/312]), and gynecologic oncology (15.2% [69/453]). Figure 1 shows the trends in internally filled positions by year for each subspecialty. The odds of a position being filled internally remained similar across the two match year cohorts after controlling for subspecialty (adjusted odds ratio 1.01, 95% CI, 0.81–1.26).

**Table 1.** Rates of Internally Filled Fellowship Positions by Subspecialty Before and After 2020

Subspecialty Type	All Match Years (2017–2022)	Before 2020 (2017–2019)	After 2020 (2020–2022)	*P*
All	409/1,910, 21.4 (19.6–23.3)	190/892, 21.3 (18.6–24.0)	219/1,018, 21.5 (19.0–24.0)	.91
GYO	69/453, 15.2 (11.9–18.5)	32/207, 15.5 (910.5–20.4)	37/246, 15.0 (10.6–19.5)	.90
MFM	210/805, 26.1 (23.1–29.1)	98/373, 26.3 (21.8–30.7)	112/432, 25.9 (21.8–30.1)	.91
REI	77/340, 22.6 (18.2–27.1)	32/166, 19.3 (13.3–25.3)	45/174, 25.9 (19.4–32.4)	.15
URPS	53/312, 17.0 (12.8–21.2)	28/146, 19.2 (12.8–25.6)	25/166, 15.1 (9.6–20.5)	.33

GYO, gynecologic oncology; MFM, maternal–fetal medicine; REI, reproductive endocrinology and infertility; URPS, urogynecology and reproductive pelvic surgery.

Data are n/N, % (95% CI) unless otherwise specified.

*McLaughlin. Internally Filled Fellowship Positions in Obstetrics and Gynecology. O&G Open 2025*.

**Fig. 2.**
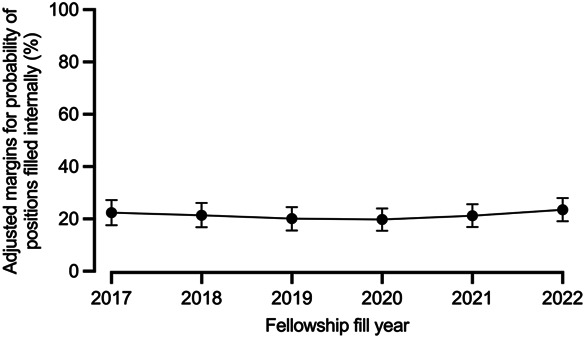
Likelihood of internally filled fellowship positions by match year for all subspecialties combined. Data presented show the probability of internally filled fellowship positions by match year in all subspecialties, with 95% CIs.

There was no significant change in the proportion of positions filled internally during the study period (adjusted marginal change [aMC] of 0.2%/year, 95% CI, −0.9 to 1.3%) (Fig. 2). Similarly, when examining the trends within the two cohorts, neither demonstrated statistical significance (2017–2019: aMC −1.1%, 95% CI, −3.7 to 1.6%; 2020–2022: aMC 1.7%, 95% CI, −0.9 to 4.4%). Additionally, comparing the change in adjusted margins between the two cohorts revealed no significant difference (delta aMC of 2.8%, *P*=.13). In a final multivariable logistic regression model that incorporated year, match year cohort, and subspecialty as independent factors, along with an interaction term between year and match year cohort, the interaction term did not attain significance (adjusted odds ratio 1.20, 95% CI, 0.93–1.56). This demonstrates that the match year cohort did not have a significant effect on the trend of internally filled positions over time.

*McLaughlin. Internally Filled Fellowship Positions in Obstetrics and Gynecology. O&G Open 2025*.

**Fig. 3.**
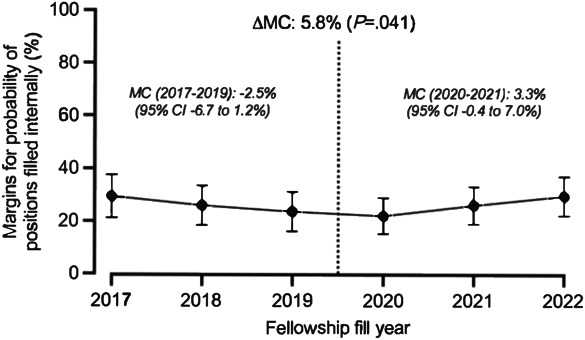
Likelihood of internally filled fellowship positions by match year in maternal–fetal medicine. Data presented show the probability of internally filled fellowship positions by match year in the maternal–fetal medicine subspecialty, with 95% CIs. MC, marginal change.

In the subanalyses conducted for each specialty, nonsignificant results were similarly observed for gynecologic oncology, reproductive endocrinology and infertility, and urogynecology and reconstructive pelvic surgery specialties. Within maternal–fetal medicine, the proportion of internally filled positions was similar between the two match year cohorts (2017–2019: MC −2.5%, 95% CI, −6.7 to 1.2%; 2020–2022: MC 3.3%, 95% CI, −0.4 to 7.0%). However, a statistically significant difference was identified when comparing the trend changes between the cohorts (delta MC 5.8%, *P*=.04) (Fig. 3).

*McLaughlin. Internally Filled Fellowship Positions in Obstetrics and Gynecology. O&G Open 2025*.

## DISCUSSION

Despite the abrupt shift from in-person to virtual interviews, the proportion of internally filled fellowship positions within obstetrics and gynecology subspecialties before and after 2020 was similar. Moreover, we did not identify a trend in this proportion over the study period, nor did we find a difference in trend when comparing the years 2017–2019 with 2020–2022. Although the largest subspecialty fellowship, maternal–fetal medicine, had a similar proportion of internally filled positions before and after 2020, there was a statistically significant shift in trend from negative to positive, suggesting that the proportion of internally filled positions is increasing relative to the time period before 2020. With this in mind, it is essential to continue the evaluation of fellowship match outcomes, including internally filled positions both through and outside of the NRMP within obstetrics and gynecology subspecialty fellowships.

Although this study is the first to explore internally filled positions in obstetrics and gynecology, other studies have observed increased rates of internally filled positions after 2020 in some procedural subspecialties. For example, one study in plastic surgery reported that 36% of positions were internally filled during the 2021 NRMP match compared with 24% in the 5 years prior.^13^ Another study involving plastic surgery residency candidates found rates of internally filled positions of 15% in 2019, 18% in 2020, and 26% in 2021.^14^ Similar data were found in two other studies, though no differences were noted in an analysis of urology applicants.^11,12,15^ However, these previously mentioned studies were limited to comparisons between the 2020–2021 match cycle, which marked the transition to virtual interviews, and the years preceding it. In contrast, our study extends the analysis to include the 3 years before and after this transition, allowing for an evaluation of trends and shifts in trend over time. It is unknown whether this difference noted in other procedural subspecialities would diminish as familiarity and comfort with virtual interviews increased over time or as in-person interviews return at some programs.

Though multiple survey-based studies have demonstrated an overall positive experience of virtual interviews within obstetrics and gynecology, there are few studies evaluating how the obstetrics and gynecology fellowship match has been affected since the transition to a virtual interview format.^3,6,8–10,16–19^ Our study suggests that the incidence of internally filled fellowship positions has remained similar despite the virtual interview format and provides objective support for continued integration of virtual interviews into the fellowship match process. Moreover, our study provides useful information for residency applicants planning to pursue obstetrics and gynecology subspecialty training as they interview and create their NRMP rank order lists. Because virtual interviews appear likely to remain the dominant format for obstetrics and gynecology subspecialty fellowships, ongoing assessment of its influence on fellowship match outcomes and equity is essential.

There was variation noted in the proportion of internally filled positions between the different subspecialties, with maternal–fetal medicine having the highest and gynecologic oncology having the lowest. Maternal–fetal medicine differs from the other obstetrics and gynecology subspecialty fellowships in several ways that may explain the higher proportion of internally filled positions, including having the largest number of fellowship positions and programs overall. Additionally, many fellowship programs accept more than one fellow per year, potentially providing more opportunities for fellowships to offer positions to internal trainees. Gynecologic oncology is also unique, in that it includes several large fellowship programs without an associated core residency program, which naturally leads to a lower overall proportion of internally filled positions. Some of those programs offer visiting rotations to residents, allowing for direct experience with applicants that may increase the likelihood of matching at that program.

Our study should be interpreted in the context of several limitations. First, we were unable to identify a small number of trainees (fewer than 1%) within filled positions due to a lack of a universal source of information in obstetrics and gynecology subspeciality fellowships.^16^ However, given the overall completeness of our data set, it is unlikely that these missing data would affect our overall study findings. Second, we included positions in military fellowship training programs to enhance the comprehensiveness and generalizability of our data set, because these positions are filled by candidates from both civilian and military residency training programs. We recognize that military programs use a separate system for matching than the NRMP, which may involve unique processes affecting the internal fill rate. We accounted for this by including clustering at the fellowship program level in our analysis; however, further studies specific to military training programs may provide additional insights into fellowship fill patterns. We also were unable to assess whether fellowship candidates had prior experience with a fellowship program outside of residency training (eg, away rotation at an institution, research project, other advanced degree), or if internally filled positions were filled within or outside of the match. Moreover, we were unable to assess the likelihood of matching into fellowship based on whether a trainee was from a residency program with a linked fellowship, because we did not have access to data on candidates who applied for fellowship but did not match.

The significant shift in trend of internally filled positions from negative to positive within maternal–fetal medicine is notable and warrants long-term follow-up. There are several other areas that need future research to provide fellowship candidates with comprehensive information about the complex application process. These include assessing the likelihood of matching into a fellowship based on whether a trainee attended a residency with a linked fellowship, the geographic distance between residency and fellowship, and if trainees attended medical school at a linked program.

The proportion of internally filled fellowship positions for gynecologic oncology, maternal–fetal medicine, reproductive endocrinology and infertility, and urogynecology and reconstructive pelvic surgery subspecialties was similar before and after 2020, when assessed both collectively and individually, despite the abrupt shift to virtual interviews. Although continued observation of these trends is warranted, this information is useful for both program directors and applicants in providing reassurance that practices in internal filling of fellowship positions remained consistent from 2017 through 2022.
